# Supplementary material for: Characterization of time-variant and time-invariant assessment of suicidality on Reddit using C-SSRS
Source: PLoS One. 2021 May 17;16(5):e0250448. doi: 10.1371/journal.pone.0250448 (PMC8128252; doi:10.1371/journal.pone.0250448)
Supplement: Attachment — Submitted filename: PONE-D-20-23705_Response_to_Reviewers_2.pdf [file pone.0250448.s003.pdf]

Characterization of Time-variant and Time-invariant Assessment of Suicidality on Reddit using C-SSRS (Summary of Changes)

We thank the reviewers again for their constructive feedback that has greatly improved the quality of this manuscript. We have addressed the changes suggested by the reviewers. Places where we have made edits, as suggested by reviewers, are colored in **brown.**

Q1: Suicide-related terms are reported much more consistently.However, as mentioned in the O'Carroll paper, the revised term for 'completed suicide' is simply 'suicide' or 'died by suicide'. Efforts are now being made to avoid all terms with "positive" connotations such as "completed" when referring to death by suicide.
Response: We have made the change in the abstract.

Q2: I am still not sure why the paragraph from line 131 to 150 is not in the method section. This paragraph seems redundant with the paragraph from line 184 to 209 (which is in the method section).
Response: We thank the reviewer for suggesting the change. We have moved the content from line numbers starting from 131 to 150 in the Introduction section to line numbers 173 to 188 in the Materials and Methods section.

Q3: Line 177: the number of posts is not indicated, it is written "XX posts”.
Response: We have entered the number of posts in the dataset.

Q4: It would be helpful to define the category "suicide indicator" when it is mentioned that this category is excluded from the study (line 253).
Response: We have provided the definition for “Suicide Indication” in line numbers starting from 252 to 260.
